# Evaluation of *Hermetia illucens* Larvae Oil as a Dietary Substitute for Fish and Vegetable Oils in African Catfish Hybrid (*Clarias gariepinus × Heterobranchus longifilis*)

**DOI:** 10.1155/anu/4693136

**Published:** 2025-07-31

**Authors:** Robert Egessa, Anita Szűcs, László Ardó, Janka Biró, Gyöngyvér Fazekas, Éva Lengyel-Kónya, Vojislav Banjac, Strahinja Vidosavljević, Kinga Katalin Lefler, Zsuzsanna J. Sándor

**Affiliations:** ^1^Research Centre for Fisheries and Aquaculture (HAKI), Hungarian University of Agriculture and Life Sciences, Szarvas, Hungary; ^2^National Agricultural Research Organisation (NARO), Jinja, Uganda; ^3^Institute of Food Science and Technology, Hungarian University of Agriculture and Life Sciences, Budapest, Hungary; ^4^Institute of Food Technology, University of Novi Sad, Novi Sad, Serbia; ^5^Department of Aquaculture, Hungarian University of Agriculture and Life Sciences, Gödöllő, Hungary

**Keywords:** antioxidative effect, black soldier fly, catfish, fatty acid metabolism, sustainability

## Abstract

Alternative sources of dietary fish oil (FO) are necessary for the growth of the aquaculture industry. This study investigated the potential benefits of black soldier fly larvae oil (BSFLO) as a feed ingredient in the diets of African catfish hybrids (*Clarias gariepinus* × *Heterobranchus longifilis*). Three isonitrogenous and isolipidic diets—a control diet (CTR) containing a FO and rapeseed oil (RO) mixture (50:50), IO50 containing BSFLO replacing 50% of FO and RO mixture and IO100 containing 100% BSFLO—were formulated. A total of 900 fish juveniles (average initial weight: 29.1 ± 1.69 g) were randomly distributed into three dietary groups, each replicated three times and reared in a recirculation aquaculture system for 7 weeks. Results showed similar fish growth between dietary groups (final body weight: CTR, 155.27 ± 4.45 g; IO50, 162.47 ± 0.19 g; IO100, 160.87 ± 3.78 g). In addition, nutrient utilisation parameters and whole-body crude protein, lipid and ash content were similar between groups. The levels of eicosapentaenoic acid (EPA) and docosahexaenoic acid (DHA) in the whole body decreased while that of arachidonic acid (ARA) increased with IO inclusion. Similar trends in ARA and EPA levels were observed in the liver, the DHA/EPA ratio being highest in fish fed IO100 diet. The hepatic expression of *pparα* (peroxisome proliferators activated receptor alpha), *hnf4α* (hepatocyte nuclear factor 4α) and *hadh* (hydroxyacyl-CoA dehydrogenase) followed quadratic trends being lowest in fish fed IO50 and highest in fish fed IO100. The *lpl* (lipoprotein lipase, LIPA) gene was significantly upregulated in fish fed IO100. The liver malondialdehyde (MDA) content was lowest in fish fed IO50. In addition, plasma myeloperoxidase (MPO) activity linearly increased with IO inclusion. These results demonstrate that potential benefits are achieved when BSFLO is supplemented in diets of African catfish hybrids, contributing to the development of sustainable alternatives to fish and vegetable oils (VOs) in aquafeeds.

## 1. Introduction

Dietary lipids, along with proteins, carbohydrates, vitamins and inorganic elements, are important in fish nutrition and play vital roles in fish growth, reproduction and movement [1]. In fish aquaculture, fish oil (FO) has been highlighted as the main source of lipids in aquafeeds because it is highly digestible and contains essential fatty acids (EFAs) in both the *n*-3 and *n*-6 families. In FO, the content of *n*-3 EFA, such as α-linolenic acid (ALA, 18:3*n*-3), eicosapentaenoic acid (EPA) (20:5*n*-3) and docosahexaenoic acid (DHA) (22:6*n*-3) is higher than that of *n*-6 EFA, such as linoleic acid (LNA) (18:2*n*-6) and arachidonic acid (ARA) (20:4*n*-6), thereby satisfying the EFA requirements of several fish species [2]. However, with the expansion of the aquaculture industry amidst increasing costs and finite supplies of FO from capture fisheries, the need to replace FO with more sustainable sources of oil for aquafeeds has increased [3]. Investigations on the use of vegetable oil (VO) as alternatives to FO in aquafeeds provided evidence that it is possible to partially or completely replace dietary FO without compromising fish growth and welfare, as long as the EFA requirement of the species is satisfied [4].

Currently, the aquaculture industry is dominated by the use of VO as lipid source in fish feeds, which is attributed to their being more efficient, sustainable and economically viable sources [5]. However, VO contain higher concentrations of *n*-6 than *n*-3 polyunsaturated FAs (PUFAs). For instance, oil extracted from soybeans, corn or cottonseed is rich in LNA (18:2*n*-6), an *n*-6 FA. This may result in farmed fish enriched with *n*-6 PUFA at the expense of *n*-3 PUFA, thereby compromising the nutritional value of the resulting farmed fish [2, 6–8]. Moreover, for some freshwater species, it has been reported that complete replacement with lipids limited in long chain-PUFA (LC-PUFA) results in decreased performance [9, 10]. Available scientific data demonstrates that insects offer a more sustainable and profitable alternative oil source than VO and have gained legal basis for their use in aquafeeds [11].

Among the insects, the black soldier fly (BSF), *Hermetia illucens* (Diptera: Stratiomyidae), is well positioned as a better substitute for FO when compared to VO, particularly when taking into account the ecological footprint (low water and land requirements) of the insect and effects on the gut health of fish [5]. In addition, BSF has fast growth and high feed conversion ratio (FCR), with ability to utilise wastes and other by-products as food [12]. The FA profile of BSF varies according to the stage of development, type of diet and the rearing conditions. Thus, the low content of LC-PUFA, such as EPA and DHA in BSF larvae can be improved by feeding the larvae on diets enriched with LC-PUFA [13, 14]. In addition, the FA profile of BSFLO is dominated by saturated FAs (SFAs), especially the medium-chain FAs (MCFAs), such as lauric acid (LA, C12:0) with antimicrobial and anti-inflammatory properties, often representing up to 52% of the total FA content [15–18].

Investigations involving terrestrial animals and fish demonstrate that MCFAs are a readily available substrate in the energy yielding reactions, since they do not need the carnitine shuttle to enter the mitochondria. This implies that feeding fish on diets rich in MCFA would increase the amount of fat that can be efficiently oxidised. Thus, reduced fat deposition in the muscle, liver and intra-peritoneal fat in fish fed diets rich in MCFAs have been reported [8, 19]. In addition, the FA profiles of BSFLO have adequate amount of LNA and linolenic acid, considered as precursors for the biosynthesis of LC-PUFA. Thus, the low dietary ARA, EPA and DHA can in part be compensated through their endogenous biosynthesis from the dietary precursor molecules [20]. The endogenous LC-PUFA biosynthesis capacity of African catfish, *Clarias gariepinus* was demonstrated [21]. Thus, both the type of dietary lipids ingested and the ability of fish to modify the dietary lipids via the catabolic and anabolic pathways determine the FA composition of the resulting fish tissues [1]. Previously, we demonstrated the importance of BSFLO in the diet of European catfish, *Silurus glanis*, with results indicating similar growth performance and nutrient utilisation across the experimental fish groups but with the lipid metabolic reactions higher in fish fed 100% BSFLO [17]. In this paper, we present results of a first study evaluating the effect of diets supplemented with BSFLO (high in LA) on the growth, nutrient utilisation, tissue FA profile, lipid metabolism and antioxidative responses of African catfish hybrid (*Clarias gariepinus × Heterobranchus longifilis*) reared under recirculation aquaculture conditions.

## 2. Material and Methods

### 2.1. Ethics Statement

All the procedures were conducted in line with the European Union Directive 2010/63/EU on animal protection for scientific purposes and approved by the Ethical Committee of the Research Centre for Aquaculture and Fisheries (HAKI), Hungarian University of Agriculture and Life Sciences (licence no. MATESZIC/2240–1/2022). Animal suffering was minimised by using an anaesthetic, Norcaicum-Tonogen (50 mL/100 L water: Norcaicum from Magilab Ltd., Budapest, Hungary; Tonogen from Gideon Richter Ltd., Budapest, Hungary).

### 2.2. Ingredients, Experimental Diets and Feeding Trial

Three diets—a control diet (CTR) and two insect oil-based diets (IO50 and IO100)—were formulated. The CTR diet contained a mixture of FO and rapeseed oil (RO) (1:1 ratio), the IO50 diet contained 50% BSFLO replacing FO and RO and the IO100 diet contained only BSFLO (100%) (Table 1). Information on ingredients used and the feed production process is as reported in our previous publication [17] except some feed production technology changes administered in order to obtain floating feed suitable for this species. The feeding trial involved a total of 900 juvenile African catfish hybrids (average initial weight: 29.1 ± 1.69 g), randomly distributed into three dietary groups (CTR, IO50 and IO100), each replicated three times (100 fishes per replicate) and reared in a recirculation aquaculture system equipped with nine 1 m³ fibreglass tanks for 7 weeks. A total of 10 fishes from the initial stock was collected and pooled for initial whole-body composition analysis. Prior to feeding with the experimental diets, all the fishes were acclimatised to experimental conditions for 2 weeks. During this period, fishes were initially fed a basal diet, followed by incremental replacement of the basal diet with a control diet until all the basal diet was completely replaced. Water quality parameters such as temperature (25.0 ± 0.10°C), dissolved oxygen (8.00 ± 0.20 mg/L) and pH (8.34 ± 0.24) were measured regularly, while ammonia (0.130 ± 0.03 mg/L), nitrites (0.003 ± 0.00 mg/L) and nitrates (14.3 ± 7.10 mg/L) were measured on a weekly basis.

### 2.3. Sample Collection

At the end of the feeding trial, fishes were fasted for 24 h and individually weighed. Ten fishes were sampled from each tank, total length measured and fishes dissected to obtain liver weight for the calculation of condition factor (CF) and hepatosomatic index (HSI), respectively. Those liver samples were stored at −80°C for determination of fat content and FA profile of the liver. Three fishes per tank (nine fishes per dietary treatment) were sampled for whole-body proximate composition analysis. Three additional fishes per tank (nine fishes per dietary treatment) were sampled and blood collected from the caudal vein, centrifuged at 1700 rpm (CAPP CR-1730R, Nordhausen, Germany) for 20 min at 4°C and plasma stored at −20°C for analysis of biochemical and immunological parameters. The same samples of fish were dissected to obtain the liver which was stored at −80°C for analysis of antioxidant status. Additionally, whole intestines were also collected and stored in 8% formalin solution until histological processing. Four fish samples per tank (12 fishes per dietary treatment) were dissected to obtain the livers which were separately placed in 2.0 mL centrifuge tubes containing RNAlater for 1 day at 4°C, followed by storage at −20°C for gene expression analysis. After an additional 3 days of feeding, three fishes per treatment were sampled and the mid-intestine obtained for assessment of digestive enzyme activity. Additional nine fishes per tank were sacrificed to collect faeces for digestibility analyses.

### 2.4. Biochemical Analysis

Whole-body samples were pooled with three fishes per tank, while liver samples were pooled with nine fishes per tank, following the same approach used for faeces samples. Fish whole-body and diets were analysed for dry matter, crude protein, crude lipid, ash content, crude fibre and gross energy, following AOAC [22]. Whole-body samples were finely ground, frozen, lyophilised, homogenised and analysed for proximate and FA profiles whereas the diets were analysed on an 'as is' basis. Briefly, dry matter was determined by drying the samples at 105°C in an oven (AOAC, 2000; method 950.46 for water), ash content by combustion in a muffle furnace (AOAC, 2000; method 942.05 for crude ash), crude protein by the Kjeldahl method (AOAC, 2000; method 954.01 for crude protein), crude lipids by Soxhlet method (AOAC, 2000; 945.16 Soxhlet method), crude fibre by using an automatic analyser (Gerhardt Fibretherm FT12 apparatus, Gerhardt GmbH & Co. KG, Germany) and the AOAC (2000; method 962.09 for crude fibre) and gross energy by direct combustion of samples in a bomb calorimeter (model 6400, Parr Instruments, Moline, IL, USA) in accordance with the manufacturer instructions. Yttrium content was analysed by the ICP method (Thermo Scientific 6500 ICP-OES, Massachusetts, USA) via digestion with mixtures of nitric acid (R.G. 65%) and hydrogen peroxide (R.G. 30%) followed by extraction using the microwave digestion technique under high pressure (Milestone Ethos Plus, Sorisole, Italy). The protein and dry matter content of the faecal samples were determined as previously presented after lyophilisation of the pooled samples. The FA composition of insect oil, diets, homogenised and lyophilised whole fish and fresh fish livers were analysed via gas chromatography (Agilent 7890 A GC System). The extraction of lipids was performed according to the ISO 12966-2:2017, n.d. standard's ‘Rapid method'. FA methyl esters (FAME) were separated on Phenomenex Zebron ZBFAME (60 m, 0.25 mm and 0.20 µm) column with cyanopropyl stationary phase and hydrogen gas as mobile phase. Identification was done using Supelco 37 (Supelco, Bellefonte, NJ, USA) component FAME mixture as primary standard, results were given in area% equally to w% total FA.  Table 2 shows the FA composition of BSFLO and the three diets (CTR, IO50 and IO100).

### 2.5. Plasma Biochemical and Immunological Parameters

The activities of alanine aminotransferase (ALT), alkaline phosphatase (ALP), as well as the contents of glucose (GLU), total protein (TP), total cholesterol (CHO), albumin (ALB), globulin (GLOB) = TP-ALB, lipase (LIPA), amylase (AMY), creatinine (CREA) and phosphorus (PHOS) were determined using a Samsung PT10V blood analyser and the Comprehensive Plus test assays (Samsung, Seoul, Republic of Korea). Plasma total immunoglobulin levels were determined according to the method described by Sharma et al. [23], while myeloperoxidase (MPO) activity was determined following Kokou et al. [24] and the absorbances measured in a plate reader (Multiskan sky, Thermo Scientific, Vantaa, Finland).

### 2.6. Digestive Enzyme Activity

To determine the activities of LIPA, trypsin and AMY, intestine extracts were prepared by homogenising samples in ice-cold buffer (50 mM Tris buffer, 200 mM NaCl, pH 7.6), in a 1:9 (w/v) sample to buffer ratio and the homogenates centrifuged (13,000 × *g* for 10 min). The resulting supernatant was aliquoted and stored at −80°C until analysis. For each sample, enzyme activity was determined in triplicates. LIPA activity was assayed according to the method of Winkler and Stuckman [25]. Trypsin activity was determined using benzoyl-DL-arginine-*p*-nitroanilide (BAPNA) as a substrate [26], while for determination of AMY activity starch (1% w/v) as substrate [27] was used. In all cases, the enzyme activities were measured as changes in absorbance using a plate reader (Multiskan sky, Thermo Scientific, Vantaa, Finland). The TP concentration of homogenates was determined based on the biuret reaction using a protein diagnostical reagent kit (Fluitest TP, Analyticon Biotechnologies AG, Lichtenfels, Germany) according to the manufacturer's instructions and specific enzyme activities (mU/mg protein) determined.

### 2.7. Antioxidant Capacity

The liver samples were homogenised and the resulting crude extracts assayed for superoxide dismutase (SOD) activity, and the contents of malondialdehyde (MDA), reduced glutathione (GSH) and total antioxidant capacity (TAOC), using commercial assay kits, according to the manufacturer's instructions. The following kits were used: GSH kit (abx096005, Abbexa, UK), SOD assay kit (CS0009, MERCK), MDA assay kit (MAK085, MERCK) and TAOC assay kit (MAK187, MERCK).

### 2.8. Histological Assessment

Intestine samples were washed under running tap water and dehydrated in graded ethanol series (70%–90%), washed in xylene and soaked in liquid paraffin wax in an automatic tissue processor (Shandon; Citadel 2000 LE11 5RG, Thermo Fisher Scientific, Waltham, Massachusetts, USA). They were then embedded in paraffin blocks using Leica HistoCore Arcadia H equipment (Leica Biosystems, Wetzlar, Germany). While using a microtome (Leica RM 2245, Leica Biosystems, Wetzlar, Germany), 5 µm sections were then cut and two sections fixed on slides in water bath (temperature: 42–44°C, Kunz Instruments HP-3, Kunz Instruments Ab, Nynashamn, Sweden). Slides were then stained using the standard haematoxylin and eosin (H&E) staining technique (Shandon Varistain 24–4, Thermo Fisher Scientific, Waltham, Massachusetts, USA) and examined under a microscope (Nikon Eclipse 600, Auroscience Consulting Ltd., Budapest, Hungary). The length of the intestinal epithelial cells was measured in µm using ImageJ software. The images on the slide were captured using a camera (QImaging Micro Publisher 3.3, QImaging, Surrey, Canada) connected to the microscope.

### 2.9. Gene Expression Analysis

Total RNA was extracted from the liver samples using the SV total RNA Isolation system (Promega, Madison, WI, USA), according to manufacturer's instructions. The quantity of RNA was determined using a Nano-Drop spectrophotometer (NANODROP 2000, Thermo Fisher Scientific, Waltham, MA, USA). The RNA integrity was checked on 1% denaturing gel electrophoresis and its purity determined by measuring the ratio of OD at 260 nm to that at 280 nm. The cDNA was then generated from 400 ng of total RNA using LunaScript RT SuperMix Kit (New England Biolabs, Ipswich, MA, USA) following the manufacturer's protocol. The resulting cDNA was then used as a template in quantitative real time polymerase chain reaction (qPCR) to determine the expression levels of genes involved in stress and lipid metabolism using the corresponding primers (Table 3). The qPCR amplifications of samples were carried out in triplicate using a LightCycler 96 instrument and the FastStart Essential DNA Green Master qPCR kit (Roche, Switzerland). The qPCR reactions were performed in a 20 μL total reaction volume consisting of 5 μL diluted (1/20) cDNA, 1 μL (10 μM) of each primer, 3 μL nuclease free water and 10 μL qPCR master mix (2×, containing Taq DNA polymerase, uracil-DNA glycosylase and dNTPs in an optimised PCR buffer). The following conditions were used in qPCR: 95°C for 10 min followed by 45 cycles of denaturation at 95°C for 15 s, annealing at 60°C for 30 s and extension at 72 °C for 30 s. Negative controls containing no cDNA were systematically run. The specificity of the qPCR reactions was checked via melting curve analysis. The mean threshold cycle (Ct) values were calculated and the comparative CT method (2^*−ΔΔCT*^ method) used to calculate the relative expression of genes [29]. The fold change was then calculated by dividing the gene expression level of individual fish fed IO50 or IO100 diets by the mean expression level of fish fed the CTR diet. The efficiencies of qPCR reactions were determined using the formula: Efficiency (%) = (10 ^(−1/slope)^−1) × 100 and values as high as 104.2% were obtained.

### 2.10. Calculations

Growth performance, feed efficiency and body indices were calculated using the following formulae [30]:

  $$\begin{array}{c} \text{Survival rate }\left(\text{\%}\right)=100\times \;\left(\text{number of survived fish}/\text{numbers of stocked fish}\right), \end{array}$$

  $$\begin{array}{c} \text{Weight gain }\left(\text{\%}\right)=100\times \left(\text{final weight }\left(g\right)-\text{initial weight}\left(g\right)\right)/\text{initial weight }\left(g\right), \end{array}$$

  $$\begin{array}{c} \text{Specific growth rate }\left(\text{\%}/\text{day}\right)=100\times \;\left[\ln\left(\text{final body weight}\right)-\ln\left(\text{initial body weight}\right)\right]/\text{number of feeding days}, \end{array}$$

  $$\begin{array}{c} \text{Feed conversion ratio }\left(\text{FCR}\right)\;=\text{total weight of dry feed given }\left(g\right)/\text{total wet weight gain }\left(g\right), \end{array}$$

  $$\begin{array}{c} \text{Protein retention }\left(\text{\%}\right)=100\times \;\left(\text{final protein content of fish biomass}-\text{initial protein content of fish biomass}\right)/\text{protein intake}, \end{array}$$

  $$\begin{array}{c} \text{Protein efficiency ratio }\left(\text{PER}\right)=\left(\text{final weight }\left(g\right)-\text{initial weight }\left(g\right)\right)\;/\text{protein intake }\left(g\right), \end{array}$$

  $$\begin{array}{c} \text{Fat retention }\left(\text{\%}\right)=100\times \left(\text{final fat content of fish biomass}-\text{initial fat content of fish biomass}\right)/\text{fat intake}, \end{array}$$

  $$\begin{array}{c} \text{Condition factor }\left(g/{\text{cm}}^{3}\right)=100\;\times \text{fish weight }\left(g\right)/\left[\text{body length }\left(\text{cm}\right)\right]3, \end{array}$$

  $$\begin{array}{c} \text{Hepatosomatic index }\left(\text{\%}\right)=100\times \;\left(\text{liver weight}/\text{body weight}\right). \end{array}$$

The lipid quality indices, such as atherogenic, thrombogenic and polyene indices and the apparent digestibility index of fishes were determined using the following formulae [28, 30]:

  $$\begin{array}{c} \text{Atherogenic index }=\;\left[C12:0+\left(4\;\times C14:0\right)\;\left.+C16:0\right)\right]/\;\left(\text{Total }n-6\text{ PUFA }+\text{ Total }n-3\text{ PUFA }+\text{Total MUFA}\right), \end{array}$$

  $$\begin{array}{c} \text{Thrombogenic index }=\;\left(C14:0\;+C16:0\;+C18\right)/\;\left[\left(0.5\;\times \text{Total MUFA}\right)|+|\left(0.5\;\times \text{ Total }n-6\text{ PUFA}\right)|\;|+|\;|\left(3\;\times \text{ Total }n-3\text{ PUFA}\right)|+|\left(\text{Total }n-3\text{ PUFA}/\text{Total }n-6\text{ PUFA}\right)\right], \end{array}$$

  $$\begin{array}{c} \text{Polyene index }\left(\text{PI}\right)=\left(C20:5+C22:6\right)/C16:0. \end{array}$$

Apparent digestibility coefficient of the diets:

  $$\begin{array}{c} {\text{ADC}}_{\text{diet}}=\left[1\;-\left(\left[{\text{Y}}_{\text{diet}}/{\text{ Y}}_{\text{faeces}}\right]\times \left[{\text{D}}_{\text{faeces}}/{\text{D}}_{\text{diet}}\right]\right)\right]\times 100. \end{array}$$

Where Y_diet_ is the dietary yttrium level, Y_faeces_ is the faeces yttrium level, D_diet_ is the dietary nutrient level and D_faeces_ is the faeces nutrient level.

### 2.11. Statistical Analysis

Statistical analyses were performed in R software (version 4.2.3) and results considered significant at 95%. Normality and homogeneity of variances were checked and normalisation applied when necessary. One-way ANOVA was used to assess the differences between dietary treatments (CTR, IO50 and IO100), followed by Tukey's post-hoc test to indicate the magnitude of differences between the means. To determine if the data followed a linear or quadratic model in response to insect oil, orthogonal polynomial contrast analysis was performed.

## 3. Results

### 3.1. Fish Growth and Nutrient Utilisation

In all the experimental trials, fish growth was over four-fold of the initial body weight. Fish grew from an initial average body weight of 29.13 ± 0.86 g to a final average body weight of 159.54 ± 3.08 g during the 7 weeks feeding period. There were no statistical differences in the growth performance (final weight, weight gain and specific growth rate), nutrient utilisation (feed conversion ratio, protein efficiency ratio, protein retention and fat retention), body indices (CF and HSI) and survival of fish fed the control (CTR) and experimental diets (IO50 and IO100) at the end of the feeding periods (Table 4).

### 3.2. Whole Body Composition

The whole-body proximate composition of fish fed the CTR and experimental diets with different lipid sources is presented in Table 5. There were no statistical differences in the whole-body crude protein, crude lipid and ash between the dietary groups. However, the whole-body moisture content was significantly different between the dietary groups and linearly increased in the direction of fish fed insect oil-based diets.

The whole-body FA profile indicated no statistical difference in the total saturated FA (SFA) between the dietary groups (Table 6). The w% of SFA, such as LA (C12:0) and myristic acid (C14:0) statistically differed between the dietary groups and linearly increased with IO fraction in the diets (Table 6). In addition, whole-body w% of MUFA and *n*-3 PUFA as well as EPA (C20:5*n*-3) and DHA (C22:6*n*-3), significantly decreased with increase in dietary IO fraction (Table 6). The whole-body MUFA content was dominated by oleic acid (C18:1*n*-9), which also determined the trend in MUFA content (Table 6). In addition, whole-body w% of total PUFA and w% of *n*-6 PUFA were significantly different between the dietary groups and linearly increased with increase in IO fraction of the diet. LNA (C18:2*n*-6) content significantly differed between the fish groups and linearly increased with increasing IO fraction of the diets. Other *n*-6 PUFAs including γ-linolenic acid ([GLNA], C18:3*n*-6), dihomo-γ-linolenic acid (DGLNA, C20:3*n*-6) and ARA, C20:4*n*-6 linearly increased with increase in IO fraction of the diets (Table 6).

### 3.3. Liver FA Profiles

The total lipid content of the liver was not statistically different between fish groups fed the CTR and IO-based diets (IO50 and IO100) (Table 7). The contents of total SFA, MUFA and PUFA were also similar across dietary groups. Among the SFAs in the liver, significant differences between the dietary groups were observed in the w% of C12:0, C14:0 and C22:0 which also linearly increased in the direction of increasing dietary IO fraction (Table 7). However, the w% of LA (C12:0) in the fish livers (CTR, 0.09%; IO50, 0.63% and IO100, 1.42%) were much lower than those of the corresponding diets (CTR, 0.34%; IO50 diet, 11.46% and IO100 diet, 22.94%). Similar observations were obtained for myristic acid (C14:0)—the w% in the liver was lower than that of the corresponding diets for all dietary fish groups (Tables 1 and 6). On the contrary, the liver samples contained higher contents of C16:0 and C18:0 (Table 7) than in the corresponding diets (Table 2). The *n*-6 PUFA content was non-significant between the dietary fish groups, but linearly increased with increase in IO fraction of the diets.

Among the MUFAs, only 14:1*n*-5, 15:1*n*-5, 18:1*n*-9*t*, 20:1*n*-9 and 22:1*n*-9 significantly differed between the dietary groups and linearly increased (14:1*n*-5, 15:1*n*-5 and 18:1*n*-9*t*) or decreased (20:1*n*-9 and 22:1*n*-9) with increase in IO fraction in the diets. The w% of gondoic acid (C20:1*n*-9) did not statistically differ between dietary groups, but linearly decreased with increase in IO fraction in the diet (Table 7). The decrease in the w% of oleic acid (C18:1*n*-9) in the liver of fish fed IO-based diets was not significant. However, the w% of 18:3*n*-6 (γ-linolenic acid, GLNA), 20:3*n*-6 (dihomo-γ-linolenic acid, DGLNA) and 20:4*n*-6 (ARA) were significantly higher in the livers of fish fed IO-based diets than in fish fed the control diet (Table 7). Among the *n*-3 PUFAs, only the w% of EPA (C20:5*n*-3) showed significant difference between the dietary groups and linearly decreased with increase in IO fraction in the diets. The liver DHA (C22:6*n*-3) content showed no statistical difference between the dietary groups. However, the DHA/EPA ratio significantly differed between the dietary fish groups, being higher in fish fed IO100 diet than in fish fed IO50 diet (Table 7).

### 3.4. Nutritional Quality Indices

The nutritional quality of the lipids in fish fed the CTR, IO50 and IO100 diets was assessed by calculating the atherogenic index (AI), thrombogenic index (TI) and polyene index (PI). The AI, TI and PI of the whole-body and liver samples were statistically similar between the dietary groups (Tables 6 and 7). However, AI of the liver increased linearly with increase in insect oil fraction of the diets. The AI was in the range of 0.21–0.53 in the whole body and 0.44–0.57 in the liver. The TI ranged from 0.20 to 0.70 in the whole body and 0.87 to1.23 in the liver while PI was in the range of 0.11 – 0.85 in the whole body and 0.02–0.12 in the liver.

### 3.5. Digestive Enzyme Activities and Apparent Digestibility Coefficients (ADC)

The hydrolytic activities of AMY, LIPA and trypsin were not statistically different between the dietary fish groups (Table 8). In addition, the ADC values of crude protein and dry matter were similar between the dietary fish groups.

### 3.6. Plasma Biochemistry

Table 9 shows results on plasma biochemical parameters of fish fed the CTR and IO-based diets (IO50 and IO100). Among all the parameters, only the content of GLU indicated a significant difference between the dietary groups, being highest in fish fed IO50 and lowest in fish fed the IO100 diet, exhibiting a quadratic trend. In addition, a linear increase in MPO activity with increase in dietary IO inclusion was observed.

### 3.7. Liver Antioxidant Capacity

**Figure 1:**
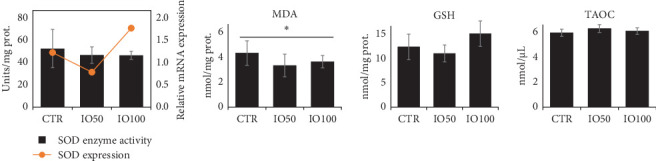
The effects of insect oil (BSFLO) on the expression and activity of SOD enzyme, as well as the contents of MDA, GSH and TAOC in the fish liver. Data presented as mean ± SD. *⁣*^*∗*^ Indicates significant results.

Among the antioxidant parameters examined (Figure 1), only the MDA content significantly varied between the dietary groups, with the lowest value recorded in the fish fed IO50 diet. The hepatic expression of *sod1* gene and the activity of SOD enzyme, a marker of oxidative stress, was similar between the dietary fish groups.

### 3.8. Intestinal Histology

**Figure 2:**
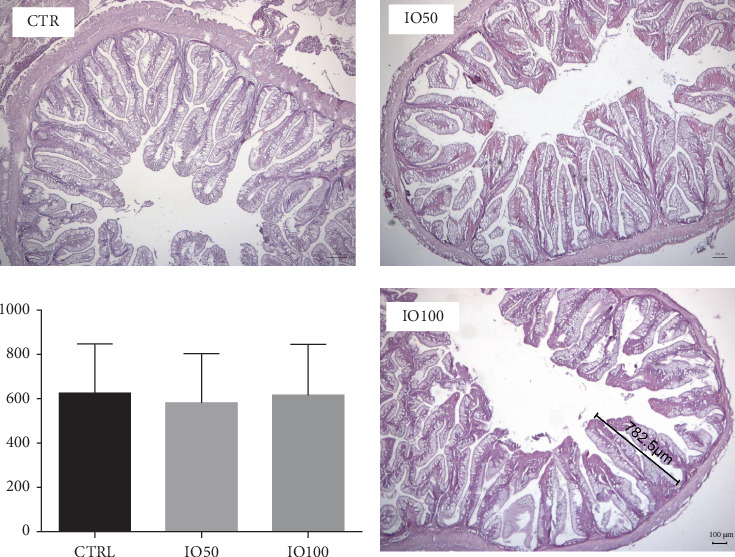
Histomorphology of intestinal villi of African catfish hybrid fed a control (CTR) diet (H&E, 40×) and insect oil-based diets (IO50 and IO100 both, H&E, 40×).

The intestinal histomorphology showed an orderly arrangement of the villi, with no significant differences in the size or number of villi in all the dietary groups (Figure 2). The microvilli projections were densely and closely positioned. Across the dietary fish groups, the epithelial cells were rich in large and evenly distributed goblet cells. The tips of the villi were regularly rounded, with no pathological changes or cell proliferation observed. The lamina propria and the epithelial layer remained connected. No pathological differences in length were observed in the cells of the epithelial layer. Any visible differences in the sections may be attributed to the characteristics of samples taken from different segments of the intestinal tract.

### 3.9. Expression of Lipid Metabolism Related Genes

**Figure 3:**
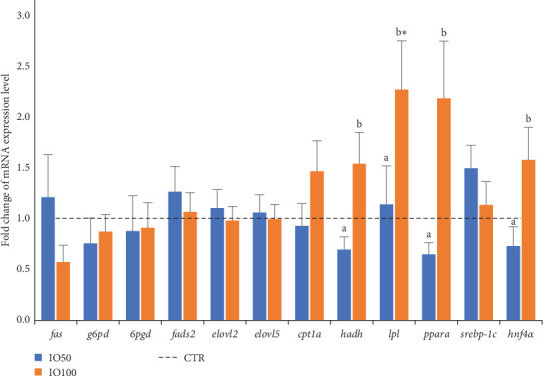
The fold change of mRNA expression level (mean ± SE, *n* = 12) of genes involved in elongation and desaturation, de novo fatty acid biosynthesis, β-oxidation and transcriptional regulation in the livers of juvenile African catfish hybrids fed the control (CTR) and experimental diets (IO50 and IO100). The fold change was calculated by dividing the mRNA expression level of individual fish fed the IO50 or IO100 diets by the mean mRNA expression level of fish fed the CTR diet. The dotted line at 1.0 expression level represents that of the control. Fold change values >1 indicate an increased mRNA expression level in the IO50 or IO100 fed fish and vice versa. For any given gene, different letters (a, b) denote statistically significant differences in the mRNA expression level between IO50 and IO100 groups, while “*⁣*^*∗*^” denote statistically significant differences in the mRNA expression level between the insect oil-based group (IO50 or IO100) and the CTR group.

To understand the effect of dietary insect oil on liver lipid metabolism, genes involved in LC-PUFA biosynthesis (*fads2*, *elovl2* and *elovl5*), de novo FA synthesis (*fas*, *g6pd* and *6gpd*), FA oxidation (*cpt1a* and *hadh*), triacylglycerol (TAG) metabolism (*lpl* gene), and transcriptional regulators of genes of lipid metabolism (*pparα*, *srebp-1c* and *hnf4α*) were studied. The results are presented as fold change of gene expression level in fish fed IO50 and IO100 diets relative to those fed the CTR diet (Figure 3). The dietary inclusion of insect oil did not affect the expression of *fas*, *g6pd*, *6pgd*, *fads2*, *elovl2*, *elovl5* and *srebp-1c*, (Figure 3). However, significant differences between the dietary groups were observed in the expression of *hadh*, *lpl*, *hnf4α* and *pparα*. The differences in expression of *hadh*, *hnf4α* and *pparα*, were mainly between fish fed IO50 and those fed IO100 and followed quadratic trends—the lowest mRNA expression level occurring in the fish fed IO50 and the highest levels in fish fed IO100. The *lpl* gene encoding lipoprotein LIPA was significantly upregulated in fish fed IO100 diet (Figure 3).

## 4. Discussion

The current study indicated that total replacement of a mixture of FO and RO with BSFLO in practical diets of juvenile African catfish hybrid does not negatively affect fish growth performance and nutrient utilisation. The major difference between the three experimental diets used in this study was the lipid sources and therefore different FA composition. Different oils are known to differently affect fish species, size and body metabolism [31–33]. Replacement of the FO/RO mixture with BSFLO mainly increased the content of saturated FAs as BSFLO was rich in saturated FAs, such as LA (C12:0), myristic acid (C12:0) and palmitic acid (C16:0). The BSFLO is naturally high in SFA especially MCFA [16, 17] and in this study, over 83% of the FA content of BSFLO was SFA, dominated by LA (51.2% of total FA). It is this high SFA that makes BSFLO different from other lipid sources, such as VOs. The effects of diets rich in MCFA have been studied in various fish species [16–36]. Despite the variation in SFA content of the diets, no alterations were observed in the growth of African catfish hybrid of this study. All the diets promoted high growth rates (FBW, WG and SGR), similar to results reported for European catfish (*Silurus glanis*) fed similarly formulated diets for 8 weeks [17], stripped catfish, *Pangasianodon hypophthalmus*, fed BSFLO, FO and moringa oil [37] and Jian carp, (*Cyprinus carpio* var. Jian), where growth (FBW and SGR) and feed intake were not affected when fish was fed diets containing up to 100% BSFLO for 59 days [19]. Juvenile *Onychostoma macrolepis* fed diets with up to 50% BSFLO replacing FO presented similar WG, SGR and feed intake, which were affected when fish was fed diets with 100% BSFLO, caused by reduced feed intake [36].

African catfish (*Clarias gariepinus*) fed 50% BSFL meal (BSFLM) presented higher WG, WG%, SGR and PPV than groups fed fish meal or 25% and 75% BSFLM, but with no effect on feed intake and survival [38]. In red hybrid tilapia (*Oreochromis* sp.) fed diets with up to 100% BSF prepupae oil replacing FO, higher growth (FBW and WG) and lowest FCR were recorded in the group fed 25% prepupae oil when compared to groups fish fed 75% and 100% prepupae oil, with no effect on palatability [35]. The high survival and stimulation of similar growth rates in African catfish hybrid of this study indicated that supplementation of diets with BSFLO did not affect feed intake and palatability since all feed given was consumed. European catfish, stripped catfish and Atlantic salmon fed control FO based diets and groups fed BSFLO based diets, presented similar levels of whole-body protein, lipid and moisture between dietary groups [16, 17, 37]. Similarly, the whole-body crude protein, lipid and ash levels of African catfish hybrid of this study did not differ between the dietary groups except for moisture content which was slightly higher in the group fed IO50 than those fed the CTR diet.

Generally, the lipid composition of fish tissues is known to largely match that of the diets. With the exception of ARA level which increased with IO inclusion, the trends in levels of other FA in whole body closely matched those of the diets. Thus, the tissue FA composition structure is determined by the dietary FA content, as well as the feed intake, growth rate and duration of feeding [39]. In this study, replacing FO with BSFLO in the diets of African catfish hybrid did not result in liver lipid deposition. Such results were also reported in a number of studies utilising BSFLO as a lipid source in the diets of fish species [12, 17, 37]. The balance between lipolytic and lipogenic processes affects fat deposition in tissues. A comparison of the level of LA in the liver with levels in the corresponding diets indicated a much lower content in the liver than in the diet, suggesting that LA was oxidised to provide energy [40]. The expression level of *cpt1a* gene encoding a protein that facilitates entry of long chain FAs (LCFAs) into the mitochondrial matrix for β-oxidation [41], was not significantly influenced by BSFLO inclusion in the diets, but overall was increased in the group fed IO100 diet. Nevertheless, the expression levels of *lpl* and *hadh* involved in TAG hydrolysis and β-oxidation respectively, were significantly increased. Thus, increased expression of *cpt1a*, *lpl* and *hadh* in IO100 group indicated that fish utilised LCFA in the β-oxidation process as well. However, the highly reduced content of LA in the livers of fish fed the IO-based diets suggested that MCFAs served as the primary energy source of energy. This is because MCFAs can pass directly through the mitochondrial membrane without the need for the carnitine shuttle unlike the LCFA which require cpt1a enzyme [40]. Significant upregulation of *cpt1a* was reported in studies utilising BSFLO oil as a lipid source for other fish species [17, 18, 36, 37].

The hepatic LC-PUFA biosynthesis genes *fads2*, *elovl2* and *elovl5* were not affected by the inclusion of BSFLO in the diets of African catfish hybrids similar to results reported for juvenile European catfish, juvenile *Onychostoma macrolepis* and rainbow trout (*Oncorhynchus mykiss*) [17, 36, 42]. However, liver FA composition showed significant differences in the levels of *n*-6 PUFAs including γ-linolenic acid (C18:3*n*-6), dihomo-γ-linolenic acid (DGLNA) (C20:3*n*-6) and ARA (C20:4*n*-6), with highest values recorded in fish fed IO100, despite the low supplies of these FA in IO100 diet. Such results suggested that the desaturation and elongation reactions occurred, being more directed towards *n*-6 LC-PUFA than *n*-3 LC-PUFA of the EPA or DHA type. Similar results in liver ARA levels were observed in juvenile black seabream [43] and European catfish [17], highlighting the essential physiological role of ARA in the generation of eicosanoids which promote inflammation [44]. The increased liver DHA/EPA ratio in fish fed IO100 was due to a decrease in EPA and increase in DHA level with BSFLO inclusion, which could be a consequence of desaturation of EPA and selective retention of DHA or retention of DHA and selective catabolism of EPA. Irrespective of the primary mechanism for the increased liver DHA/EPA ratio, the lack of significant differences in the expression of *fads2*, *elovl2* and *elovl5* between the dietary groups suggested that selective retention of DHA could have played a major role. The expression of genes of transcription factors, *pparα* and *hnf4α* playing pivotal roles in dietary FA-mediated effects [45, 46], differed between the groups. In juvenile European catfish and Jian carp (*Cyprinus carpio* var. Jian), inclusion of BSFLO had no effect on the expression of *pparα*, but a significant upregulation of *hnf4α* was observed in European catfish [17, 19].

Among the plasma biochemical and immunological parameters, only the GLU content and MPO activity were influenced by dietary BSFLO inclusion. These results are different from those obtained for European catfish fed similarly formulated diets [17]. In addition, the activities of ALP and the levels of ALB, GLOB and ALB/GLOB ratio significantly differed in groups of European catfish [17], contrary to the results of this study. In other studies, the levels of TP, ALB, CHOL, GLU as well as ALB/GLOB ratio were not affected [37, 19, 47]. Rainbow trout fed BSFLO-based diets relative to FO indicated differences in the GLU and PHOS content between the dietary groups [34]. MPO which catalyses the reaction of hydrogen peroxide with chlorine to produce hypochlorous acid with a strong resistance to micro-organisms, can be used as a marker for the assessment of fish health [48, 49]. The linear increase in MPO activity with BSFLO inclusion indicated influence of BSFLO on the non-specific immune function of African catfish hybrid. Among the antioxidation parameters evaluated, only MDA level was influenced by the diets. The MDA, an end-product of lipid peroxidation which is capable of damaging cell structure and function was low in fish fed BSFLO-based diets probably due to reduced dietary PUFA levels [50, 51].

## 5. Conclusions

The growth of fish was not affected by the inclusion of BSFLO in the diets of African catfish hybrid. The FA profiles of whole-body and liver largely reflected that of the diets, but deviations were observed in the levels of ARA and DHA. Liver LC-PUFA biosynthesis genes *fads2*, *elovl2* and *elovl5* were not affected, and a positive effect on the expression of *cpt1a*, *lpl* and *hadh* in fish fed IO100 was observed. The increase in plasma MPO activity with dietary content of BSFLO indicated a positive effect on the non-specific immune function. The MDA and GSH levels indicated that BSFLO-based diets could reduce oxidative stress. Therefore, BSFLO can be used as an alternative lipid source in the diet of African catfish hybrid without negatively impacting their growth, lipid metabolism, antioxidant capacity and immunity.

## Figures and Tables

**Table 1:** Dietary composition (g/kg of diet) of the experimental diets.

Ingredient (g/kg)	Diets		
	CTR	IO50	IO100
Fish oil (FO)^a^	25	12.5	0
Rapeseed oil (RO)^b^	25	12.5	0
Insect oil (BSFLO)^c^	0	25	50
Constant ingredients^d^	950	950	950

^a^FF Skagen, Skagen, Denmark.

^b^Victoria oil, Šid, Serbia.

^c^Agroloop, Budapest, Hungary.

^d^A detailed description of the experimental diets has been published by Egessa et al. [17].

**Table 2:** Fatty acid composition of BSFLO and experimental diets (w% of total FA) fed to African catfish hybrid.

Fatty acid	BSFLO	Diets		
		CTR	IO50	IO100
C12:0	51.19	0.34	11.46	22.94
C13:0	0.04	0.01	0.02	0.02
C14:0	12.04	2.27	4.18	6.22
C14:1*n*-5	0.46	0.08	0.19	0.26
C15:0	0.32	0.22	0.22	0.24
C15:1*n*-5	0.12	0.06	0.04	0.01
C16:0	15.12	13.59	14.15	14.85
C16:1*n*-7	2.76	2.86	2.75	2.68
C17:0	0.27	0.16	0.18	0.21
C17:1*n*-7	0.20	0.22	0.20	0.18
C18:0	2.76	3.56	3.63	3.74
C18:1*n*-9*t*	0.30	0.12	0.12	0.21
C18:1*n*-9*c*	0.57	34.07	26.18	17.94
C18:2*n*-6*c*	11.49	26.65	25.28	23.50
C18:2*n*-6*t*	0.05	0.16	0.10	0.02
C18:3*n*-6	0.01	0.08	0.06	0.04
C18:3*n*-3	0.09	4.38	3.51	2.55
C19:0	0.04	0.08	0.07	0.06
C20:0	0.45	0.35	0.27	0.19
C20:1*n*-9	0.08	3.04	1.94	0.83
C20:2*n*-6	0.02	0.23	0.15	0.10
C20:3*n*-6	0.02	0.06	0.05	0.04
C20:4*n*-6 (ARA)	0.04	0.31	0.25	0.19
C20:5*n*-3 (EPA)	0.03	2.56	1.63	0.73
C22:0	0.02	0.25	0.19	0.13
C22:1*n*-9	0.02	0.50	0.32	0.14
C22:2*n*-6	—	0.02	0.02	0.01
C22:6*n*-3 (DHA)	0.01	3.44	2.26	1.07
C23:0	0.01	0.03	0.03	0.02
C24:0	0.13	0.10	0.12	0.07
C24:1*n*-9	0.07	0.12	0.06	0.19
∑SFA	83.68	21.03	34.90	49.30
∑MUFA	4.57	41.07	31.78	22.45
∑PUFA	11.75	37.89	33.32	28.25

*Note:* t/c trans and cis isomers of FA.Values are presented based on duplicate analyses. C4:0, C6:0, C8:0, C10:0 and C11:0 detected and used in calculation of total SFA but not included in the table.

Abbreviations: ARA, arachidonic acid; BSFLO, black soldier fly larvae oil; DHA, docosahexaenoic acid; EPA, eicosapentaenoic acid; MUFA, monounsaturated fatty acid; PUFA, polyunsaturated fatty acid; SFA, saturated fatty acid.

**Table 3:** List of primers for quantitative real-time PCR (qPCR) analysis of the expression of genes involved in and lipid metabolism in African catfish hybrid.

Gene	Code	Primer sequence (5′–3′)	GenBank no. /reference
Peroxisome proliferators activated receptor alpha	*pparα*	F: TATTCCAAGGGTCGTTGGCG R: CTGGATCACTCATTGGACTCAG	XM_053508188.1

Sterol regulatory element binding protein-1	*srebp-1*	F: CCTGGAGGTAAAATCGAAGAGTG R: CGCTTGTCCCCTAGCTTCTC	XM_053514460.1

Hepatocyte nuclear factor 4α	*hnf4α*	F: GTCAGGTAGCTGAGAATGCGT R: CTGGCGAGGAGTCTGTGCTC	XM_053483068.1

Glucose 6-phosphate dehydrogenase	*g6pd*	F: AGATGTGGGAAAGCGCTGAA R: GACACGTACCACCAGTTCGT	XM_053482785.1

6-Phosphogluconate dehydrogenase	*6pgd*	F: CTTTACCACCGCACTGTCCT R: CTGGCCAGGATTTGAGAGCA	XM_053482564.1

Fatty acid synthase	*fas*	F: TGACGGCTACACCCCATCTA R: TCCCATCACACACCTCATGC	XM_053511065.1

Elongation of very long-chain fatty acid 2	*elovl2*	F: GCAGTACTCTGGGCATTTGTC R: GGGACATTGGCGAAAAAGTA	[21]

Elongation of very long-chain fatty acid 5	*elovl5*	F: ACTCACAGTGGAGGAGAGC R: GGAATGGTGGTAAACGTGCA	[21]

Fatty acyl desaturase 2	*fads2*	F: TCCTATATGCTGGAACTAATGTGG R: AGGATGTAACCAACAGCATGG	[21]

Hydroxyacyl-CoA dehydrogenase	*hadh*	F: GGCTCCAGGAACAAGTCAGG R: TTTGGAACCATGACCTCGCT	XM_053490732.1

Carnitine palmitoyl transferase 1a	*cpt1a*	F: CGCATGTTCAACACCAGTCG R: AGCAGGCGTCCGTCATAAAA	XM_053493295.1

Lipoprotein lipase	*lpl*	F: GCGAGACACAAACCAGGGTA R: AGCTGCCGTGCATTTTAAGC	XM_053486752.1

Superoxide dismutase 1	*sod1*	F: GGCCTAGTGCCTGGTTTACA R: CAGATCTCCAACATGCCTGA	XM_053516133.1

Elongation factor 1 alpha	*elf1a*	F: CCTTCAACGCTCAGGTCATC R: TGTGGGCAGTGTGGCAATC	[28]

**Table 4:** Growth performance, nutrient utilisation and body indices of African catfish hybrid fed the CTR and experimental diets (IO50 and IO100) for 7 weeks.

Parameters	Dietary groups			ANOVA *p*-value	Polynomial contrast	
	CTR	IO50	IO100		Linear *p*-value	Quadratic *p*-value
IBW (g)	28.81 ± 1.26	30.31 ± 2.27	28.27 ± 1.78	0.574	0.786	0.344
FBW (g)	155.27 ± 4.45	162.47 ± 0.19	160.87 ± 3.78	0.229	0.195	0.229
WG (%)	439.11 ± 8.11	437.61 ± 39.63	469.77 ± 22.53	0.488	0.335	0.520
SGR (%/day)	3.56 ± 0.04	3.66 ± 0.14	3.64 ± 0.03	0.493	0.345	0.511
FCR	0.75 ± 0.02	0.74 ± 0.03	0.73 ± 0.01	0.836	0.586	0.923
PER	3.10 ± 0.10	3.15 ± 0.14	3.17 ± 0.07	0.786	0.535	0.868
CF (g cm^−3^)	0.81 ± 0.00	0.83 ± 0.01	0.81 ± 0.01	0.266	0.994	0.131
HSI (%)	1.04 ± 0.05	1.04 ± 0.14	1.13 ± 0.14	0.702	0.504	0.668
FR (%)	122.03 ± 8.04	119.11 ± 13.89	111.72 ± 8.43	0.503	0.273	0.773
PR (%)	46.28 ± 1.73	46.18 ± 1.60	46.62 ± 0.95	0.924	0.768	0.625
SR (%)	91.5 ± 3.50	89.5 ± 1.50	89.0 ± 0.00	0.960	0.735	0.406

*Note:* Data expressed as mean ± SD.

Abbreviations: CF, condition factor; FBW, final body weight; FCR, feed conversion ratio; FR, fat retention; HSI, hepatosomatic index; IBW, initial body weight; PER, protein efficiency ratio; PR, protein retention; SGR, specific growth rate; SR, survival rate; WG, weight gain.

**Table 5:** Whole-body proximate composition (% wet weight) of African catfish hybrid fed with experimental diets (means ± SD, *n* = 3).

Treatment	Initial	Dietary groups			ANOVA *p*-value	Polynomial contrast	
		CTR	IO50	IO100		Linear *p*-value	Quadratic *p*-value
Moisture	77.01	72.43 ± 0.61^a^	73.47 ± 0.33^b^	73.29 ± 0.16^a,b^	**0.046**	**0.045**	0.083
Crude protein	13.55	14.66 ± 0.07	14.42 ± 0.13	14.51 ± 0.10	0.066	0.114	0.059
Crude fat	6.51	8.92 ± 0.30	8.90 ± 0.70	8.57 ± 0.58	0.703	0.467	0.718
Ash	1.87	2.34 ± 0.15	2.46 ± 0.17	2.59 ± 0.26	0.368	0.174	0.975

*Note:* Statistical *p*-values refer to differences between dietary groups (CTR, IO50 and IO100). Values in the same line with different superscript letters are significantly different (*p* < 0.05). The bolded numbers refers when significant differences occures.

**Table 6:** Whole body fatty acid composition (% of total fatty acids) and lipid quality indices of African catfish hybrid fed with different experimental diets (mean ± SD, *n* = 3).

Fatty acid	Initial	Dietary groups			ANOVA *p*-value	Polynomial contrast	
		CTR	IO50	IO100		Linear *p*-value	Quadratic *p*-value
12:0	0.13	0.46 ± 0.35^a^	3.40 ± 0.20^b^	5.58 ± 0.16^c^	**<0.001**	**<0.001**	0.074
14:0	1.97	1.34 ± 0.14^a^	2.04 ± 0.20^b^	2.44 ± 0.22^b^	**0.001**	**<0.001**	0.320
14:1*n*-5	0.04	0.03 ± 0.00^a^	0.05 ± 0.00^b^	0.06 ± 0.00^c^	**<0.001**	**<0.001**	0.225
15:0	0.17	0.15 ± 0.01	0.12 ± 0.03	0.11 ± 0.02	0.117	0.052	0.549
16:0	9.76	20.46 ± 0.17	12.26 ± 7.71	12.87 ± 4.76	0.278	0.128	0.790
16:1*n*-7	4.81	2.57 ± 0.10^b^	2.74 ± 0.08^b^	2.30 ± 0.07^a^	**0.001**	**0.006**	**0.002**
17:0	0.08	0.16 ± 0.01	0.10 ± 0.06	0.10 ± 0.04	0.242	0.128	0.495
18:0	3.20	8.31 ± 0.31	4.84 ± 3.15	5.14 ± 1.77	0.204	0.143	0.288
18:1*n*-9	42.87	37.91 ± 0.52^c^	34.53 ± 1.05^b^	25.23 ± 1.70^a^	**<0.001**	**<0.001**	**0.013**
18:2*n*-6	16.22	16.50 ± 0.09^a^	29.60 ± 11.10^a,b^	38.96 ± 6.57^b^	**0.017**	**0.006**	0.410
18:3*n*-6	0.39	0.29 ± 0.02^a^	0.40 ± 0.03^b^	0.51 ± 0.03^c^	**<0.001**	**<0.001**	0.927
18:3*n*-3	3.33	1.94 ± 0.03^c^	1.77 ± 0.09^b^	1.11 ± 0.04^a^	**<0.001**	**<0.001**	**0.013**
20:0	0.13	0.29 ± 0.00	0.16 ± 0.09	0.12 ± 0.04	0.051	**0.024**	0.336
20:1*n*-9	4.06	2.77 ± 0.04^c^	2.08 ± 0.11^b^	1.11 ± 0.09^a^	**<0.001**	**<0.001**	0.062
20:2*n*-6	0.32	0.29 ± 0.02^a^	0.28 ± 0.02^a^	0.21 ± 0.01^b^	**0.001**	**<0.001**	**0.032**
20:3*n*-6	0.83	0.66 ± 0.04^a^	0.83 ± 0.05^b^	0.85 ± 0.06^b^	**0.006**	**0.004**	0.071
20:4*n*-6	0.93	0.53 ± 0.03^a^	0.69 ± 0.09^b^	0.71 ± 0.03^b^	**0.016**	**0.009**	0.124
20:3*n*-3	0.12	0.07 ± 0.01	0.06 ± 0.01	0.04 ± 0.00	**0.011**	**0.005**	0.184
20:5*n*-3	2.72	1.00 ± 0.04^c^	0.76 ± 0.06^b^	0.38 ± 0.04^a^	**<0.001**	**<0.001**	0.074
22:0	0.06	0.18 ± 0.01^b^	0.08 ± 0.06^a,b^	0.05 ± 0.02^a^	**0.011**	**0.005**	0.276
22:1*n*-9	0.31	0.19 ± 0.02^c^	0.14 ± 0.03^b^	0.06 ± 0.01^a^	**<0.001**	**<0.001**	0.561
22:5*n*-3	1.31	0.55 ± 0.04	0.61 ± 0.11	0.57 ± 0.37	0.948	0.889	0.778
22:6*n*-3	5.62	2.98 ± 0.09^c^	2.23 ± 0.26^b^	1.33 ± 0.12^a^	**<0.001**	**<0.001**	0.563
24:0	0.06	0.07 ± 0.00^b^	0.05 ± 0.02^a,b^	0.04 ± 0.01^a^	**0.042**	**0.017**	0.478
24:1*n*-9	0.53	0.30 ± 0.01^c^	0.20 ± 0.06^b^	0.11 ± 0.01^a^	**0.002**	**<0.001**	0.837
SFA	15.57	31.43 ± 0.31	23.04 ± 11.42	26.45 ± 6.90	0.343	0.214	0.453
MUFA	52.63	43.77 ± 0.54^c^	39.74 ± 1.00^b^	28.88 ± 1.85^a^	**<0.001**	**<0.001**	**0.008**
PUFA	31.80	24.80 ± 0.23	37.22 ± 11.28	44.67 ± 6.84	**0.049**	**0.019**	0.661
*n*-6 PUFA	18.69	18.27 ± 0.17^a^	31.80 ± 11.26^b^	41.23 ± 6.65^c^	**0.017**	**0.006**	0.410
*n*-3 PUFA	13.11	6.53 ± 0.14^c^	5.42 ± 0.19^b^	3.44 ± 0.26^a^	**<0.001**	**<0.001**	**0.021**
DHA/EPA	2.07	2.99 ± 0.13	2.95 ± 0.42	3.47 ± 0.05	0.087	0.062	0.181
PUFA/SFA	2.04	0.79 ± 0.00	1.99 ± 1.15	1.84 ± 0.86	0.198	0.118	0.362
AI	0.21	0.38 ± 0.02	0.33 ± 0.18	0.39 ± 0.11	0.782	0.946	0.502
TI	0.20	0.59 ± 0.00	0.39 ± 0.27	0.46 ± 0.18	0.343	0.214	0.453
PI	0.85	0.19 ± 0.01	0.30 ± 0.13	0.15 ± 0.06	0.167	0.266	0.115

*Note:* Statistical *p*-values refer to differences between dietary groups (CTR, IO50 and IO100). Values in the same line with different superscript letters are significantly different (*p* < 0.05). The fatty acids C4:0, C6:0, C8:0, C10:0 and C11:0, were detected and used in the calculation of total SFA but not included in the table. The bolded numbers refers when significant differences occures.

Abbreviations: AI, atherogenic index; PI, polyene index; TI, thrombogenic index.

**Table 7:** Liver fatty acid composition (w% of total FA) and total lipid content (on wet weight basis) of African catfish hybrid fed with experimental diets fed for 7 weeks (means ± SD, *n* = 3).

FA	Dietary groups			ANOVA *p*-value	Polynomial contrast	
	CTR	IO50	H100		Linear *p*-value	Quadratic *p*-value
Total lipid (g/100 g)	8.18 ± 0.66	9.11 ± 2.59	8.00 ± 0.19	0.967	0.909	0.823
12:0	0.09 ± 0.03^a^	0.63 ± 0.21^b^	1.42 ± 0.28^c^	**<0.001**	**<0.001**	0.428
14:0	1.15 ± 0.06^a^	1.61 ± 0.08^b^	2.18 ± 0.03^c^	**<0.001**	**<0.001**	0.198
14:1*n*-5	0.03 ± 0.01^a^	0.04 ± 0.00^a,b^	0.06 ± 0.00^b^	**0.005**	**0.002**	0.839
15:0	0.08 ± 0.01	0.10 ± 0.03	0.11 ± 0.01	0.291	0.146	0.628
15:1*n*-5	0.01 ± 0.00^a^	0.02 ± 0.01^a^	0.03 ± 0.01^b^	**0.005**	**0.002**	0.205
16:0	24.55 ± 1.79	23.93 ± 1.91	23.41 ± 0.47	0.682	0.402	0.964
16:1*n*-7	3.33 ± 0.24	3.34 ± 0.23	3.26 ± 0.03	0.863	0.678	0.749
17:0	0.14 ± 0.01	0.15 ± 0.01	0.17 ± 0.01	0.056	0.022	0.723
17:1*n*-7	0.20 ± 0.02	0.22 ± 0.02	0.21 ± 0.03	0.464	0.702	0.254
18:0	11.60 ± 0.76	11.69 ± 0.35	12.16 ± 0.43	0.456	0.260	0.636
18:1*n*-9*t*	0.23 ± 0.02^a^	0.27 ± 0.01^b^	0.29 ± 0.01^b^	**0.007**	**0.003**	0.394
18:1*n*-9*c*	42.42 ± 2.16	41.50 ± 0.90	39.21 ± 0.22	0.066	**0.028**	0.502
18:2*n*-6*t*	0.24 ± 0.04	0.26 ± 0.02	0.40 ± 0.22	0.477	0.245	0.888
18:2*n*-6*c*	6.01 ± 0.40	6.87 ± 1.21	6.38 ± 0.60	0.482	0.571	0.299
18:3*n*-6	0.26 ± 0.04^a^	0.34 ± 0.02^b^	0.42 ± 0.01^c^	**0.002**	**<0.001**	0.968
18:3*n*-3	0.46 ± 0.01	0.41 ± 0.22	0.31 ± 0.05	0.423	0.215	0.799
19:0	0.08 ± 0.02	0.07 ± 0.01	0.07 ± 0.02	0.761	0.677	0.560
20:0	0.20 ± 0.02	0.25 ± 0.11	0.19 ± 0.01	0.967	0.886	0.615
20:1*n*-9	2.56 ± 0.04^b^	2.32 ± 0.16^a,b^	2.03 ± 0.11^a^	**0.004**	**0.001**	0.778
20:2*n*-6	0.56 ± 0.05	0.63 ± 0.10	0.56 ± 0.03	0.422	0.981	0.207
20:3*n*-6	1.49 ± 0.16^a^	1.90 ± 0.15^b^	2.31 ± 0.02^c^	**<0.001**	**<0.001**	0.996
20:4*n*-6 (ARA)	1.17 ± 0.14^a^	1.56 ± 0.18^b^	2.04 ± 0.09^c^	**<0.001**	**<0.001**	0.675
20:5*n*-3 (EPA)	0.53 ± 0.01^b^	0.46 ± 0.07^a,b^	0.29 ± 0.01^a^	**0.001**	**<0.001**	0.201
22:0	0.06 ± 0.01^b^	0.07 ± 0.01^b^	0.10 ± 0.00^a^	**0.001**	**<0.001**	0.076
22:1*n*-9	0.19 ± 0.01^b^	0.17 ± 0.04^b^	0.10 ± 0.01^a^	**0.007**	**0.003**	0.234
22:2*n*-6	0.01 ± 0.02	0.02 ± 0.01	0.03 ± 0.01	0.236	0.103	0.952
22:6*n*-3 (DHA)	1.65 ± 1.30	0.82 ± 1.18	1.82 ± 0.05	0.377	0.476	0.236
23:0	0.03 ± 0.03	0.05 ± 0.02	0.03 ± 0.00	0.650	0.957	0.374
24:0	0.06 ± 0.01	0.08 ± 0.01	0.08 ± 0.01	0.053	0.022	0.465
24:1*n*-9	0.14 ± 0.08	0.20 ± 0.05	0.24 ± 0.02	0.165	0.069	0.844
SFA	38.51 ± 2.37	38.65 ± 1.98	40.01 ± 0.58	0.567	0.350	0.647
MUFA	49.11 ± 2.48	48.08 ± 1.10	45.44 ± 0.09	0.067	0.029	0.493
PUFA	12.38 ± 1.71	13.27 ± 1.99	14.55 ± 0.67	0.304	0.140	0.865
*n*-3 PUFA	2.64 ± 1.32	1.68 ± 1.19	2.42 ± 0.02	0.526	0.805	0.287
*n*-6 PUFA	9.74 ± 0.77	11.58 ± 1.64	12.13 ± 0.66	0.087	**0.040**	0.439
DHA/EPA	3.07 ± 2.41^b^	1.82 ± 2.60^a^	6.25 ± 0.40^c^	**<0.001**	**<0.001**	**0.002**
PUFA/SFA	0.32 ± 0.06	0.35 ± 0.07	0.36 ± 0.02	0.645	0.365	1.000
AI	0.48 ± 0.05	0.51 ± 0.04	0.56 ± 0.01	0.085	**0.031**	0.581
TI	1.00 ± 0.11	1.07 ± 0.14	1.04 ± 0.03	0.701	0.602	0.521
PI	0.09 ± 0.05	0.05 ± 0.05	0.09 ± 0.00	0.504	0.947	0.261

*Note:* Statistical *p*-values refer to differences between dietary groups (CTR, IO50 and IO100). Values in the same line with different superscript letters are significantly different (*p* < 0.05). The fatty acids C4:0, C6:0, C8:0, C10:0 and C11:0, were detected and used in the calculation of total SFA but not included in the table. The bolded numbers refers when significant differences occures.

Abbreviations: AI, atherogenic index; PI, polyene index; TI, thrombogenic index.

**Table 8:** Digestive enzyme activity (mU/mg prot.) of African catfish hybrid fed different diets and the ADC for protein and dry matter of the diets.

Treatment	Dietary groups			ANOVA *p*-value
	CTR	IO50	IO100	
Enzyme^a^				
Amylase	25.47 ± 2.36	28.65 ± 0.46	26.81 ± 3.47	0.344
Lipase	15.51 ± 2.88	16.57 ± 2.50	15.11 ± 0.82	0.725
Trypsin	269.96 ± 16.59	303.48 ± 33.21	274.44 ± 14.42	0.233
ADC^b^				
Dry matter	68.55 ± 4.63	68.47 ± 2.94	64.57 ± 7.24	0.903
Crude protein	83.45 ± 2.25	83.98 ± 1.99	81.15 ± 4.59	0.784

^a^Mean ± SD, *n* = 9.

^b^Mean ± SD, *n* = 3.

**Table 9:** Plasma biochemical and immunological parameters (mean ± SD) of African catfish hybrid fed with CTR and experimental diets (IO50 and IO100) for eight and 7 weeks respectively.

Parameter	Dietary groups			ANOVA *p*-value	Polynomial contrasts	
	CTR	IO50	IO100		Linear *p*-value	*p*-Value quadratic
ALT	27.00 ± 4.94	21.50 ± 3.33	24.83 ± 6.61	0.209	0.477	0.106
ALP	39.00 ± 6.69	43.33 ± 5.57	36.33 ± 4.27	0.126	0.423	0.061
CHOL	129.50 ± 14.07	125.00 ± 11.17	116.50 ± 15.03	0.270	0.117	0.771
LIPA	<20	≤20	<20	—	—	—
AMY	23.67 ± 7.44	21.17 ± 7.81	23.17 ± 9.39	0.859	0.918	0.594
GLU	125.33 ± 26.21^a,b^	147.67 ± 26.63^b^	103.50 ± 17.26^a^	**0.019**	0.132	**0.014**
CREA	0.27 ± 0.10	0.33 ± 0.19	0.33 ± 0.14	0.667	0.442	0.655
CA	10.97 ± 0.26	11.03 ± 0.29	11.30 ± 0.32	0.144	0.066	0.502
TBIL	0.21 ± 0.05	0.22 ± 0.07	0.21 ± 0.03	0.931	0.873	0.736
PHOS	8.10 ± 0.36	8.17 ± 0.54	8.45 ± 0.61	0.473	0.256	0.679
TP	2.45 ± 0.16	2.33 ± 0.23	2.36 ± 0.12	0.524	0.433	0.416
ALB	<1.0	<1.0	<1.0	—	—	—
GLOB	2.32 ± 0.19	2.30 ± 0.26	2.28 ± 0.11	0.959	0.776	1.000
IG	1.58 ± 0.31	1.70 ± 0.36	1.80 ± 0.24	0.486	0.324	0.501
MPO	0.87 ± 0.30	1.01 ± 0.15	1.11 ± 0.18	0.110	**0.039**	0.845

*Note:* Alanine transaminase, ALT (U/L); alkaline phosphatase, ALP (U/L); cholesterol, CHOL (mg/dL); lipase, LIPA (U/L); amylase, AMY (U/L); glucose, GLU (mg/dL); creatinine, CREA (mg/dL); calcium, CA (mg/dL); total bilirubin, TBIL (mg/dL); phosphorus, PHOS (mg/dL); total protein, TP (g/dL); albumin, ALB (g/dL); globulin, GLOB (g/dL); immunoglobulin, IG (g/dL); myeloperoxidase, MPO (OD450nm). The bolded numbers refers when significant differences occures.

## Data Availability

Data will be made available upon request.
